# Supplementary material for: Diverse synaptic and dendritic mechanisms of complex spike burst generation in hippocampal CA3 pyramidal cells
Source: Nat Commun. 2019 Apr 23;10:1859. doi: 10.1038/s41467-019-09767-w (PMC6478939; doi:10.1038/s41467-019-09767-w)
Supplement: Supplementary file 1 — Supplementary Information [file 41467_2019_9767_MOESM1_ESM.pdf]

Supplementary Information

Diverse synaptic and dendritic mechanisms of complex spike burst generation in hippocampal CA3 pyramidal cells

Raus Balind et al.

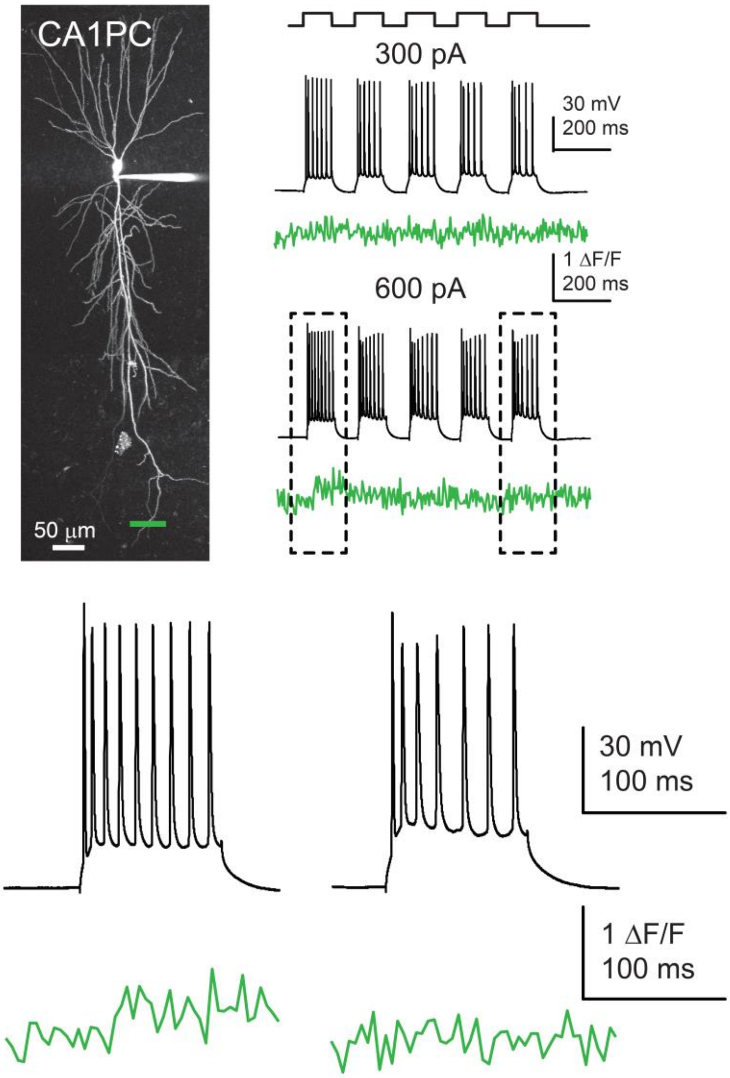

Supplementary Figure 1. Typical regular firing pattern of a representative CA1 PC to 300 pA and 600 pA I_{inj} series. Note the lack of large distal dendritic Ca^{2+} signals even with the high-affinity Ca^{2+} dye OGB-1 used.

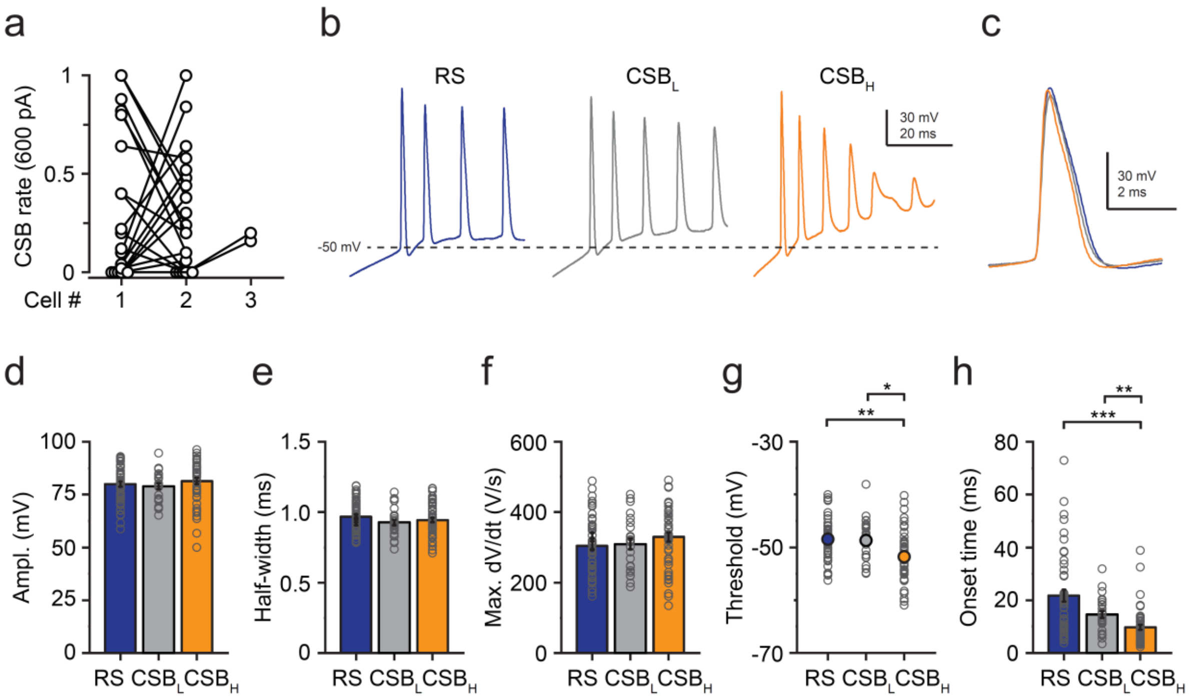

Supplementary Figure 2. a Analysis of CSB propensity in CA3PCs from individual animals. Lines connect CSB rate of cells that were measured sequentially (in different slices prepared from the same rat) under the same experimental conditions. In 50% of cases (13 out of 26) cells belonged to different CSB categories. In 5 of these 13 cases, the cells were recorded in the same CA3 subregion. **b** Representative APs evoked by 600 pA I_{inj} (part of the first pulse shown to illustrate AP properties) in a RS (left), CSB$_L$ (middle) and CSB$_H$ (right) cell. Dashed line indicates -50 mV. **c** The first APs from panel B, aligned to the onset. **d-h** Summary of the quantitative AP properties in RS, CSB$_L$ and CSB$_H$ neurons. Group data are given as mean ± s.e.m. Kruskal-Wallis test (**d**: $p=0.253$; **e**: $p=0.428$; **f**: $p=0.216$; **g**: $p=0.001$; **h**: $p<0.0001$), followed by *post hoc* multiple comparisons test if significant effect was found (**g**: RS vs. CSB$_L$: $p=1$; RS vs. CSB$_H$: $p=0.002$; CSB$_L$ vs. CSB$_H$: $p=0.018$. **h**: RS vs. CSB$_L$: $p=0.762$; RS vs. CSB$_H$: $p<0.001$; CSB$_L$ vs, CSB$_H$: $p=0.007$). Only the significant differences are indicated. RS: n=47; CSB$_L$: n=25; CSB$_H$: n=46.
*: $p<0.05$; **: $p<0.01$; ***: $p<0.001$.

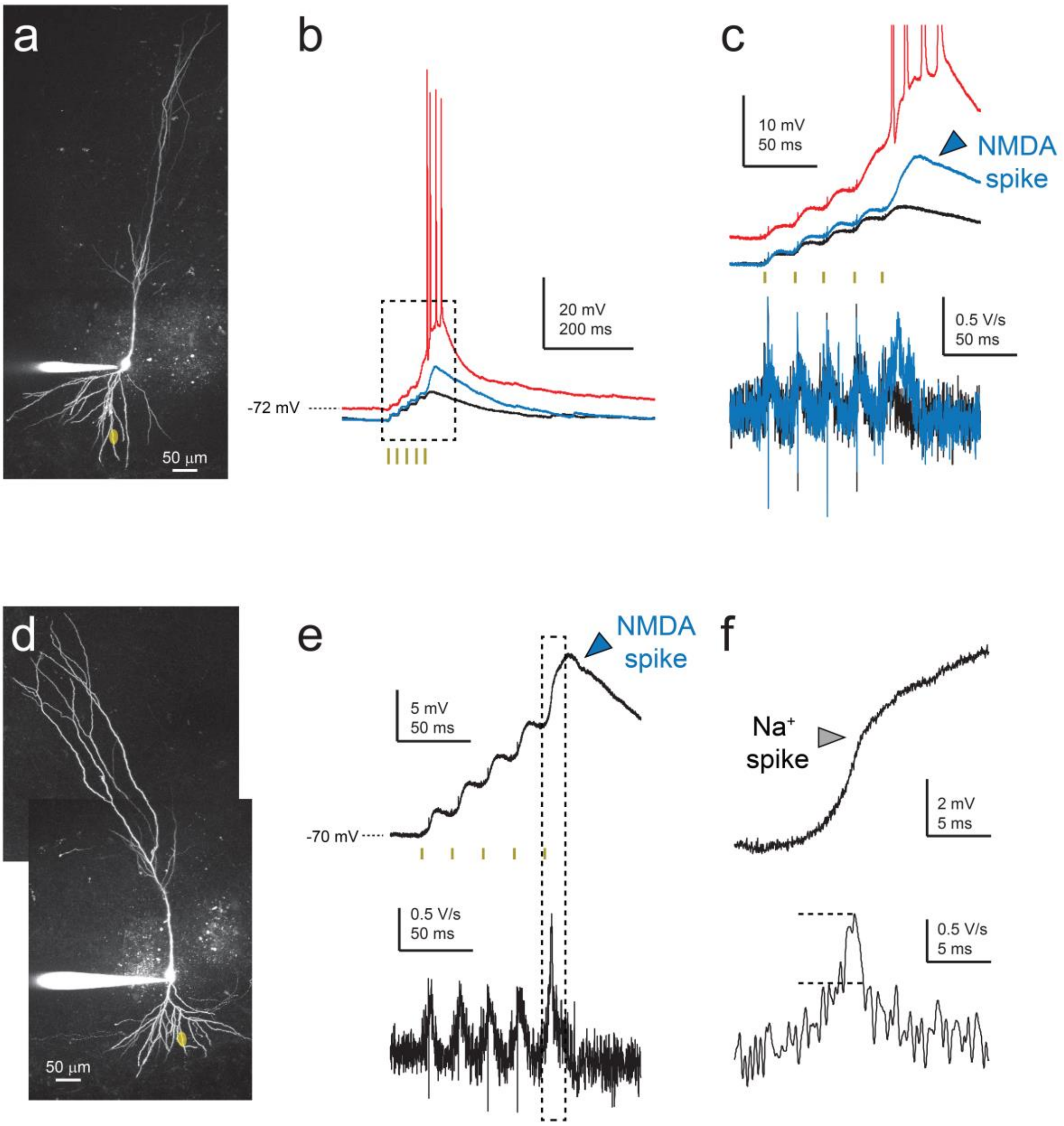

Supplementary Figure 3. Dendritic Na^+ and NMDAR-mediated spikes in two example CA3PCs. **a** Z-stack of a CA3PC with uncaging location in a basal dendrite indicated by yellow circle. **b** Somatically suprathreshold stimulation (red) by quasi-synchronous uncaging at 15 nearby spines, repeated 5x at 40 Hz. AP generation could be prevented by slight hyperpolarization, where the same stimulus produced responses with (blue) or without (black) triggering of slow dendritic spikes on the last stimulus - reminiscent of typical dendritic NMDA spikes – in an all-or-none fashion. All traces were obtained using the same uncaging stimulus settings, and are representative of several similar recordings from the same dendrite. **c** Top: traces in the dashed box from **b** are shown enlarged. Bottom: first derivative (dV/dt) of the somatically subthreshold traces. **d** Z-stack of another CA3PC with uncaging location in a basal dendrite indicated by yellow circle. **e** Top: voltage response to a similar uncaging stimulus as in **b-c**, with a stimulus strength subthreshold to AP generation. Note the large slow response on the last stimulus, indicating an NMDA spike. Bottom: corresponding dV/dt trace. **f** Trace in the dashed box from **e** is shown enlarged. Note the fast spikelet associated with an increased rate of rise on the dV/dt trace, characteristic to dendritic Na^+ spikes.

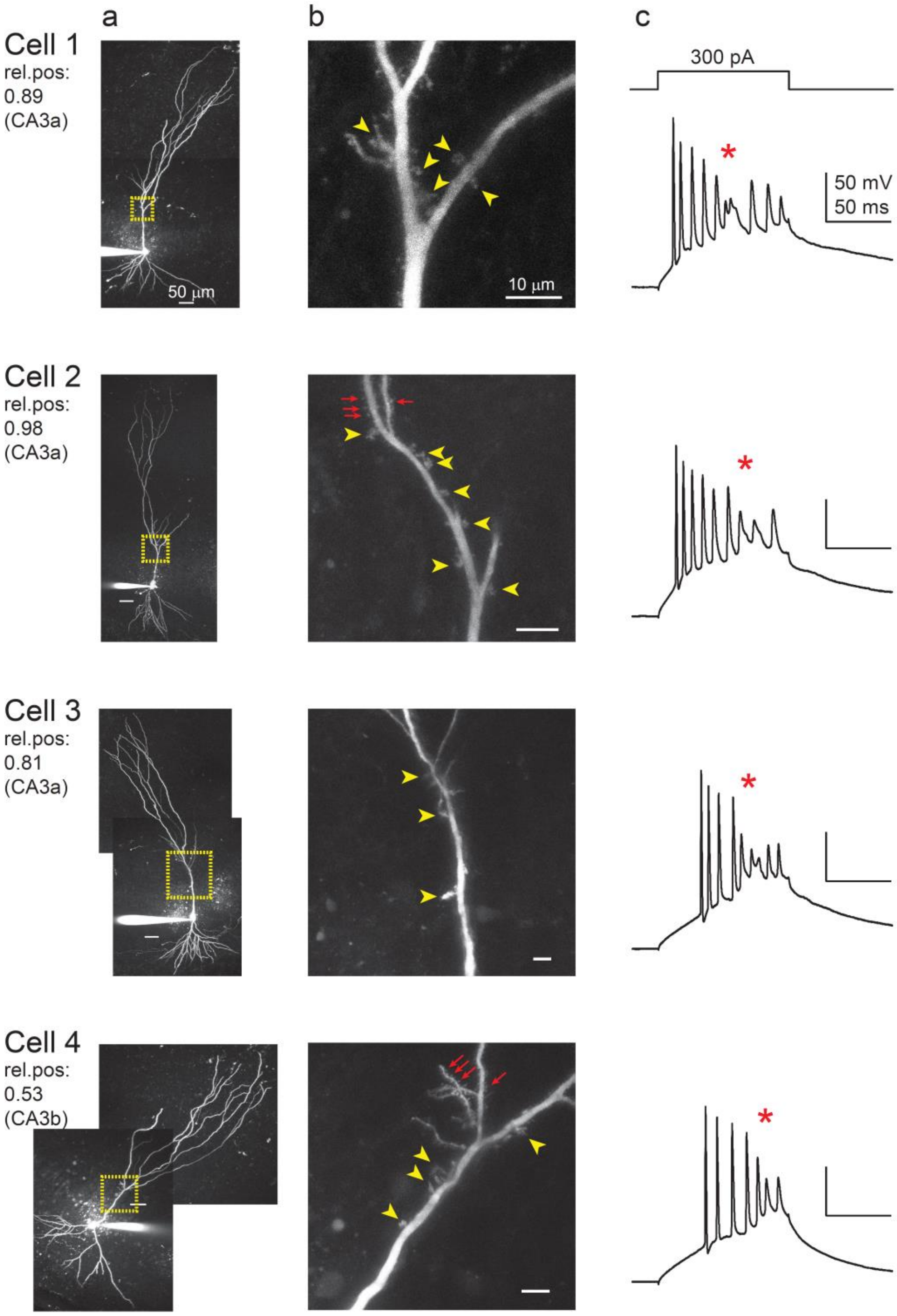

Supplementary Figure 4. Thorny CSB_H cells in deep layers of distal CA3 of adult rat hippocampus. Four example CA3PCs located at the border of, or within str. oriens in CA3a and b. Relative proximodistal position is indicated. **a** Low magnification stacks. Note the long primary apical trunk of deep cells crossing str. pyramidale. All scale bars, 50 µm. Yellow dashed boxes indicate the location of thorns near the first branching of the trunk in str. lucidum. **b** Higher magnification of the boxes in **a**. Some of the thorns (i.e. complex spines: large irregularly shaped postsynapstic structures) are indicated by yellow arrowheads. Red arrows point to a few simple spines for comparison. All scale bars, 10 µm. **c** Complex spike bursts to 300 pA somatic I_{inj} recorded in the cells shown in **a-b** (first pulse). All scale bars, 30 mV, 50 ms.

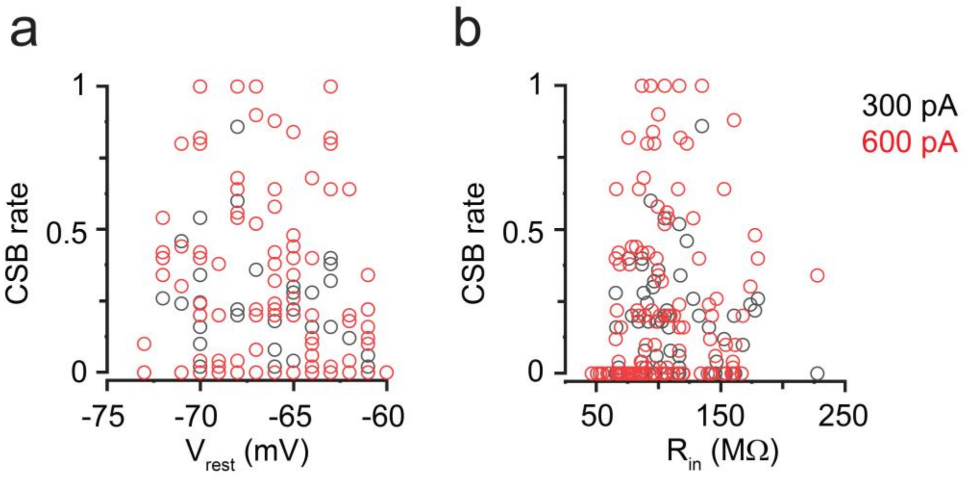

Supplementary Figure 5. Relationship between V_{rest} and CSB rate (**a**), and input resistance and CSB rate (**b**) in the whole population of CA3PCs.

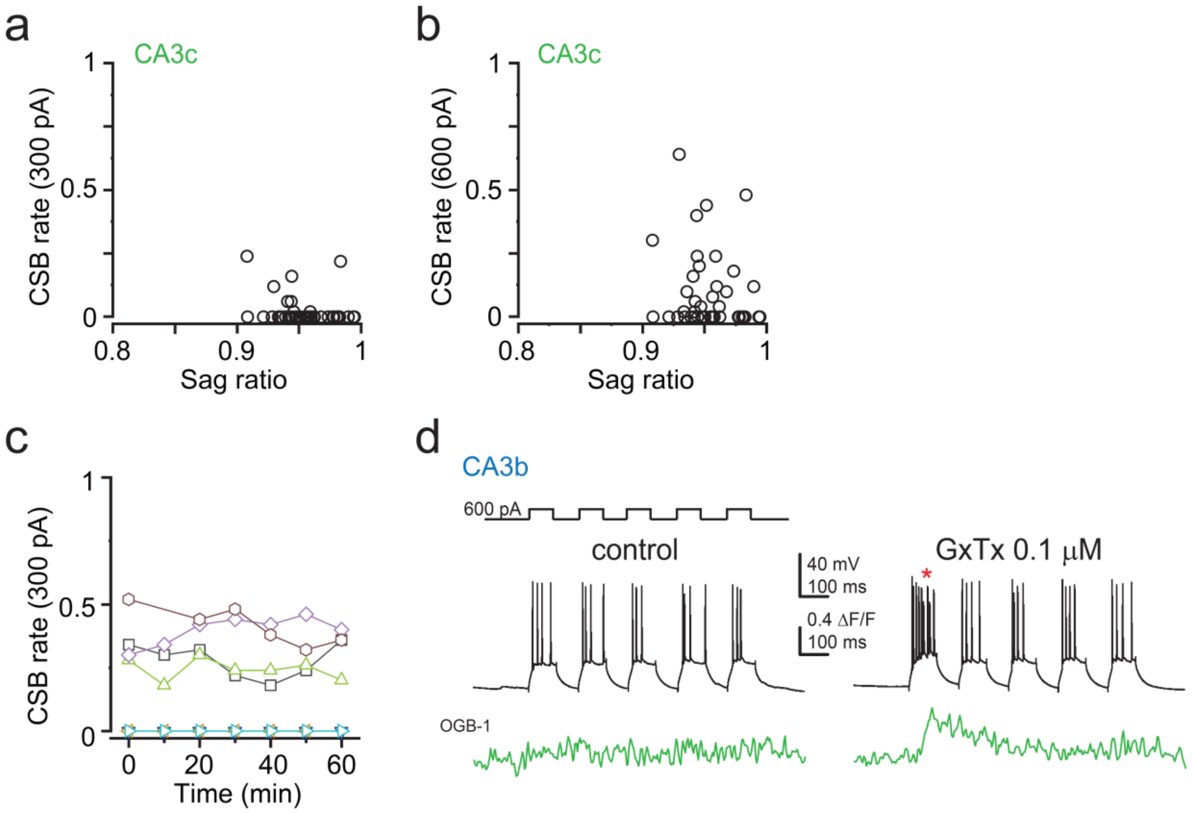

Supplementary Figure 6. Related to Figures 6-7. **a-b** Relationship of sag ratio and CSB rate (**a:** 300 pA; **b:** 600 pA) in PCs of the CA3c region. **c** CSB rate measured every 10 min under control conditions (0' vs 50' with 300 pA: n=7 (three CA3c and four CA3a cells), p=0.465; Wilcoxon test). **d** Representative experiment testing the effect of GxTx (0.1 μM) on CSB rate in a CA3b PC. CSB indicated by red asterisk.
