# Supplementary material for: Diverse synaptic and dendritic mechanisms of complex spike burst generation in hippocampal CA3 pyramidal cells
Source: Nat Commun. 2019 Apr 23;10:1859. doi: 10.1038/s41467-019-09767-w (PMC6478939; doi:10.1038/s41467-019-09767-w)
Supplement: Supplementary file 3 — Reporting Summary [file 41467_2019_9767_MOESM3_ESM.pdf]

nature research

Corresponding author(s): Judit Makara

Last updated by author(s): Mar 24, 2019

Reporting Summary

Nature Research wishes to improve the reproducibility of the work that we publish. This form provides structure for consistency and transparency in reporting. For further information on Nature Research policies, see Authors & Referees and the Editorial Policy Checklist.

Statistics

For all statistical analyses, confirm that the following items are present in the figure legend, table legend, main text, or Methods section.

n/a	Confirmed	
☐	☒	The exact sample size (n) for each experimental group/condition, given as a discrete number and unit of measurement
☐	☒	A statement on whether measurements were taken from distinct samples or whether the same sample was measured repeatedly
☐	☒	The statistical test(s) used AND whether they are one- or two-sided<br>*Only common tests should be described solely by name; describe more complex techniques in the Methods section.*
☐	☒	A description of all covariates tested
☐	☒	A description of any assumptions or corrections, such as tests of normality and adjustment for multiple comparisons
☐	☒	A full description of the statistical parameters including central tendency (e.g. means) or other basic estimates (e.g. regression coefficient) AND variation (e.g. standard deviation) or associated estimates of uncertainty (e.g. confidence intervals)
☐	☒	For null hypothesis testing, the test statistic (e.g. F, t, r) with confidence intervals, effect sizes, degrees of freedom and P value noted<br>*Give P values as exact values whenever suitable.*
☒	☐	For Bayesian analysis, information on the choice of priors and Markov chain Monte Carlo settings
☒	☐	For hierarchical and complex designs, identification of the appropriate level for tests and full reporting of outcomes
☒	☐	Estimates of effect sizes (e.g. Cohen's d, Pearson's r), indicating how they were calculated

Our web collection on statistics for biologists contains articles on many of the points above.

Software and code

Policy information about availability of computer code

Data collection	Data collection was performed using the commercially available data acquisition software system of the Ultima microscope (Prairie Technologies,Bruker).
Data analysis	Data analysis was performed using the following commercial softwares: Linescan Viewer (Prairie Technologies), ImageJ, IgorPro, Vaa3D, Statistica. Custom written macros were used for analysis in IgorPro.

For manuscripts utilizing custom algorithms or software that are central to the research but not yet described in published literature, software must be made available to editors/reviewers. We strongly encourage code deposition in a community repository (e.g. GitHub). See the Nature Research guidelines for submitting code & software for further information.

Data

Policy information about availability of data

All manuscripts must include a data availability statement. This statement should provide the following information, where applicable:

- Accession codes, unique identifiers, or web links for publicly available datasets
- A list of figures that have associated raw data
- A description of any restrictions on data availability

The data and custom code that support the findings of this study are available from the corresponding author upon reasonable request.

Field-specific reporting

Please select the one below that is the best fit for your research. If you are not sure, read the appropriate sections before making your selection.

☒ Life sciences ☐ Behavioural & social sciences ☐ Ecological, evolutionary & environmental sciences

Life sciences study design

All studies must disclose on these points even when the disclosure is negative.

Sample size	No statistical methods were used to predetermine sample sizes, but our samples are similar to or exceed those reported in previous publications and that are generally employed in the field.
Data exclusions	Criteria for inclusion of experiments based on technical and biological parameters are described in the Methods (Patch-clamp recordings, Focal electrical stimulation, Data analysis). These criteria were preestablished.
Replication	Attempts at replication were successful. The number of replicates are indicated for every experiment.
Randomization	No explicit randomization method was used, but experiments comparing different cell types were typically interleaved. Cells were assigned to experiments based on their initial CSB propensity profile. Stated in Methods
Blinding	The experimenter was aware of the experimental condition in the case of the electrophysiology-imaging experiments, because cells were assigned to experiments based on their initial CSB propensity profile. Morphological analysis was performed blind to the electrophysiological phenotype of neurons.

Reporting for specific materials, systems and methods

We require information from authors about some types of materials, experimental systems and methods used in many studies. Here, indicate whether each material, system or method listed is relevant to your study. If you are not sure if a list item applies to your research, read the appropriate section before selecting a response.

Materials & experimental systems

n/a	Involved in the study	
☐	☒	Antibodies
☒	☐	Eukaryotic cell lines
☒	☐	Palaeontology
☐	☒	Animals and other organisms
☒	☐	Human research participants
☒	☐	Clinical data

Methods

n/a	Involved in the study	
☒	☐	ChIP-seq
☒	☐	Flow cytometry
☒	☐	MRI-based neuroimaging

Antibodies

Antibodies used	We used mouse monoclonal anti-Kv2.1 (catalog 75-315, clone L80/21, RRID: AB_2315863, IgG3, 1:500, UC Davis/NIH NeuroMab Facility, Davis, CA) and Kv2.2 (catalog 75-369, clone 372B/1, RRID: AB_2315870, IgG1, 1:500, Neuromab) antibodies.
Validation	Specificity of the immunolabelings with the same Kv2.1 and Kv2.2 antibodies was previously confirmed in knockout mice (Bishop et al., 2015 J. Neuroscience).

Animals and other organisms

Policy information about studies involving animals; ARRIVE guidelines recommended for reporting animal research

Laboratory animals	7-12 week old male Wistar rats
Wild animals	The study did not involve wild animals.
Field-collected samples	The study did not involve field-collected data.
Ethics oversight	Methods were approved by the Animal Care and Use Committee of the Institute of Experimental Medicine of the Hungarian Academy of Sciences.

Note that full information on the approval of the study protocol must also be provided in the manuscript.
